# Transparent Touch: Insights From Model Systems on Epidermal Control of Somatosensory Innervation

**DOI:** 10.3389/fncel.2021.680345

**Published:** 2021-05-31

**Authors:** Chang Yin, Eric Peterman, Jeffrey P. Rasmussen, Jay Z. Parrish

**Affiliations:** Department of Biology, University of Washington, Seattle, WA, United States

**Keywords:** epidermis, somatosensory neuron, axon and dendrite development, *C. elegans*, *Drosophila*, zebrafish

## Abstract

Somatosensory neurons (SSNs) densely innervate our largest organ, the skin, and shape our experience of the world, mediating responses to sensory stimuli including touch, pressure, and temperature. Historically, epidermal contributions to somatosensation, including roles in shaping innervation patterns and responses to sensory stimuli, have been understudied. However, recent work demonstrates that epidermal signals dictate patterns of SSN skin innervation through a variety of mechanisms including targeting afferents to the epidermis, providing instructive cues for branching morphogenesis, growth control and structural stability of neurites, and facilitating neurite-neurite interactions. Here, we focus onstudies conducted in worms (*Caenorhabditis elegans*), fruit flies (*Drosophila melanogaster*), and zebrafish (*Danio rerio*): prominent model systems in which anatomical and genetic analyses have defined fundamental principles by which epidermal cells govern SSN development.

## Introduction

Why focus on these model systems? Our understanding of patterns and mechanisms of Somatosensory neuron (SSN) innervation in human skin is limited by several challenges. First, human skin exhibits remarkable diversity in its structure across anatomical locations, varying in thickness, permeability, and cellular composition. Single-cell RNA-seq (scRNA-seq) studies demonstrate the presence of multiple distinct subpopulations of fibroblasts, keratinocytes, and other dermal cells at various locations in mammalian skin (Joost et al., 2016, 2020; Cheng et al., 2018; Philippeos et al., 2018). For example, analysis of human scalp, foreskin, and trunk skin revealed eight keratinocyte subtypes that were present in varying relative proportions and exhibited significant transcriptional differences across anatomical sites (Cheng et al., 2018).

Second, in addition to differences in resident skin cells, patterns and densities of SSN innervation vary across skin types and anatomical locations. These regional specializations have been extensively characterized in the mammalian touch system, where innervation density correlates with tactile acuity (Johansson and Vallbo, 1979; Paré et al., 2002; Mancini et al., 2014). Tactile afferents densely innervate distal limbs, providing high tactile acuity, with hands and feet showing gradients in innervation (Corniani and Saal, 2020). Likewise, humans exhibit spatially distinct response properties to nociceptive stimuli, with the spatial acuity for nociceptive inputs higher on fingertips than in neighboring skin (Mancini et al., 2013).

Third, a precise accounting of the type, number, and distribution of SSNs innervating human skin is incomplete, as is the catalog of peripheral arborization patterns. Historical classifications of conduction velocity and fiber diameter undersample SSN cell type diversity, and while scRNA-seq studies are rapidly expanding the molecular taxonomy of SSNs in mice (Usoskin et al., 2015; Nguyen et al., 2017; Sharma et al., 2020), measures of SSN diversity remain understudied in humans. Likewise, until recently, peripheral arborization patterns of mammalian SSNs were largely uncharacterized. Sparse genetic labeling techniques have closed this gap in mice, with more than a dozen morphological classes of cutaneous arbors identified in recent years (Badea et al., 2012; Wu et al., 2012; Rutlin et al., 2014; Bai et al., 2015; Kuehn et al., 2019; Neubarth et al., 2020; Li and Ginty, 2014; Olson et al., 2017). Many of these arbors form specialized structures with skin cells that are essential to SSN function, underscoring the importance of skin cell contributions to SSN development.

The model systems discussed here (*C. elegans*, *Drosophila*, *D. rerio*) provide solutions to many of these problems. Chief among them, these organisms offer transparent skin and *ex utero* development that renders SSNs optically accessible, providing a direct window into SSN development *in vivo*. These systems also offer sophisticated genetic toolkits that facilitate manipulation of gene function with single-cell resolution, reagents to simultaneously and independently visualize skin cells and SSNs, and a repertoire of epidermal cells and SSNs whose developmental origins and peripheral morphologies are defined.

### C. elegans

**Figure 1:**
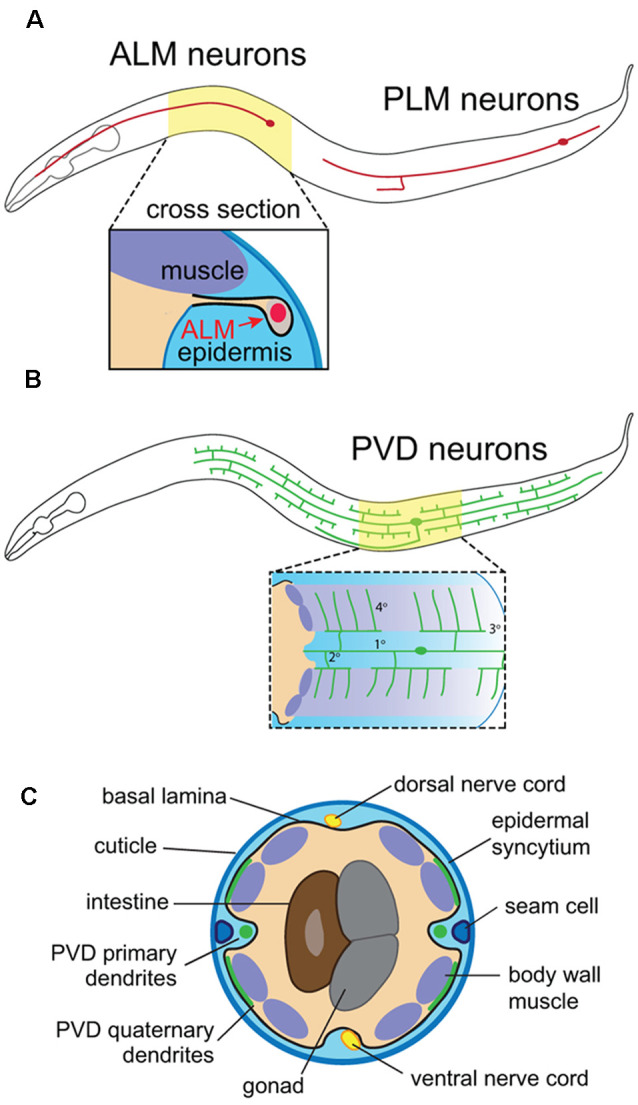
Anatomy of *C. elegans* somatosensory neurons (SSNs) and skin. **(A)** Position of the touch receptor neurons ALM and PLM, and schematic depicting epidermal ensheathment of touch receptor neurons (TRNs) shown in cross-section. **(B)** PVD neuron position and schematic depicting PVD interactions with muscle and epidermal cells. **(C)** Cross-sectional anatomy of *C. elegans* adult.

The compact nervous system, invariant lineage and morphological stereotypy of *C. elegans* neurons (Sulston et al., 1983; White et al., 1986; Corsi et al., 2015) have facilitated genetic screens for factors that influence SSN morphogenesis. *C. elegans* hermaphrodites have only 302 neurons and possess both sensory neurons that innervate the epidermal layer (also known as the hypodermis) and motor neurons that traverse the body and receive instructive epidermal cues. The touch receptor neurons (TRNs), sensory neurons PVD and FLP, and motor neurons provide instructive examples of different modes of epidermal signaling that contribute to skin innervation patterns. First, the bipolar mechanosensory TRNs ALM and PLM (anterior and posterior lateral microtubule cells) extend distinctive anterior and posterior processes, and their polarized outgrowth is controlled by epidermal cues. Epidermal cells ensheath axons of these neurons, providing insight into the developmental origin and function of this specialized epidermis-SSN interaction (Figure 1A). Second, PVD and FLP function as polymodal nociceptors (Chatzigeorgiou et al., 2010; Mohammadi et al., 2013) and elaborate highly branched dendritic arbors (Albeg et al., 2011; Figures 1B,C). PVD neurons have a stereotypic menorah-like dendritic arbor shape that branches at regular positions and in regular orientations (Smith et al., 2010), providing a sensitive and highly quantitative system for analyzing dendrite morphogenesis. These dendrites grow at the interface of muscle and epidermal cells (Oren-Suissa et al., 2010), and a series of genetic screens revealed signaling systems involving adhesive interactions with muscle and epidermal cells that shape dendrite morphogenesis (Dong et al., 2013; Salzberg et al., 2013). Finally, motor neurons run adjacent to the epidermis, from which they receive guidance cues and epidermal phagocytic activity influences synapse maintenance at the neuromuscular junction (NMJ; White et al., 1986; Cherra and Jin, 2016).

*C. elegans* skin is comprised of a simple epidermis that secretes an apical cuticle consisting of a collagenous extracellular matrix (ECM) and is surrounded on the basal surface by a basement membrane (BM; Chisholm and Hsiao, 2012; Figure 1C). This epidermis is primarily composed of a multinucleate syncytium of hypodermal cells that forms during embryonic development, prior to the peripheral innervation by sensory neurons. The epidermal primordium forms on the dorsal surface of gastrulation stage embryos, undergoes epiboly to generate an embryonic skin, and finally, epidermal cells fuse with one another to generate epidermal syncytia (Podbilewicz and White, 1994; Chisholm and Hsiao, 2012). This process is largely complete by mid-embryogenesis, yielding nine distinct hypodermal syncytia, the largest of which (hyp7) spans the majority of the animal. The adult skin additionally contains terminally differentiated seam cells, lateral hypodermal cells embedded on the apical face of hyp7 that fuse in adult worms. Of note, seam cell divisions that occur during larval stages give rise to a variety of neurons and support cells (Chisholm and Hsiao, 2012).

### Drosophila

**Figure 2:**
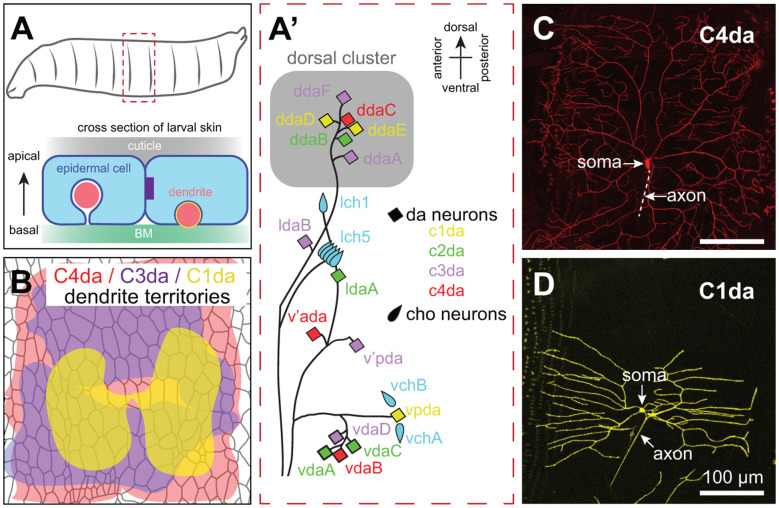
Anatomy of *Drosophila* SSNs and skin. **(A)** Organization of the larval abdominal peripheral nervous system (PNS) depicting neurons discussed in this review (left) and cross-sectional view depicting the relative position of epidermal cells, apical cuticle, basement membrane (BM), and SSN dendrites (right). Note that es neurons and subsets of md neurons are omitted from the schematic. **(B)** Peripheral territories of *Drosophila* SSNs. Schematic depicts dendritic territories of C4da (nociceptors, red), C3da (gentle touch receptors, purple), and C1da (proprioceptors, yellow) neurons and distribution of epidermal cells in a representative abdominal hemisegment. Schematic adapted from Grueber et al. (2002) and Parrish et al. (2009). **(C,D)** Confocal micrographs of representative **(C)** C4da and **(D)** C1da neurons are shown. Image credits: C4da neuron (Peng et al., 2015); C1da neuron (Lin et al., 2015) under the Creative Commons License.

The *Drosophila* larval peripheral nervous system (PNS) has served as a powerful experimental system for analysis of SSN dendrite morphogenesis, cell spacing, and dendrite-epidermis interactions that shape innervation patterns. Unlike vertebrate dorsal root ganglion (DRG) neurons, cell bodies of *Drosophila* SSNs are located in the periphery, where sensory organ precursors delaminate from the ectoderm early during embryogenesis and give rise to neurons in a highly stereotyped spatiotemporal birth order (Bodmer et al., 1989). The larval PNS is segmentally organized (Figure 2A), with each abdominal segment consisting of a fixed number of SSNs with stereotyped positions, morphologies, functional properties, and developmental trajectories (Singhania and Grueber, 2014). Thus, as in *C. elegans*, a given neuron can be reproducibly identified and assayed.

*Drosophila* embryonic/larval SSNs fall into two general types: type I and type II neurons. Type I neurons have a single unbranched dendrite and innervate external sense (es) organs or chordotonal (cho) organs; as discussed below, studies of cho neuron development have revealed roles for epidermal cues in guiding SSN migration and orienting dendrite outgrowth (Kraut and Zinn, 2004; Mrkusich et al., 2010). Type II multi dendrite (md) neurons include the dendrite arborization (da) neurons, whose highly branched dendrite arbors have been intensively studied for more than 20 years (Gao et al., 1999). While peripheral glia ensheath SSN axons and cell bodies, the glial sheaths extend only a few microns into the dendritic compartment (Yadav et al., 2019), providing a system to study direct epidermis-dendrite interactions.

Da neurons fall into four classes (Class I–IV) on the basis of larval dendrite arborization patterns (Grueber et al., 2002). These morphological classes correspond to functional types as axon laminar targeting (Grueber et al., 2007) and responses to sensory stimuli (Hughes and Thomas, 2007; Song et al., 2007; Xiang et al., 2010; Tsubouchi et al., 2012; Yan et al., 2013) correlate with dendritic morphology. Studies in *Drosophila* have been particularly instructive in identifying epidermal mechanisms that influence SSNs in a type-specific manner. Dendrites of da neurons innervate a two-dimensional territory at the basal surface of the epidermis, with arbors of different types of neurons intermingling while exhibiting distinct geometry, targeting, and occupying distinct areas (Figures 2A–D; see also Figure 4). Remarkably, each of these parameters is controlled by epidermal cues.

**Figure 3:**
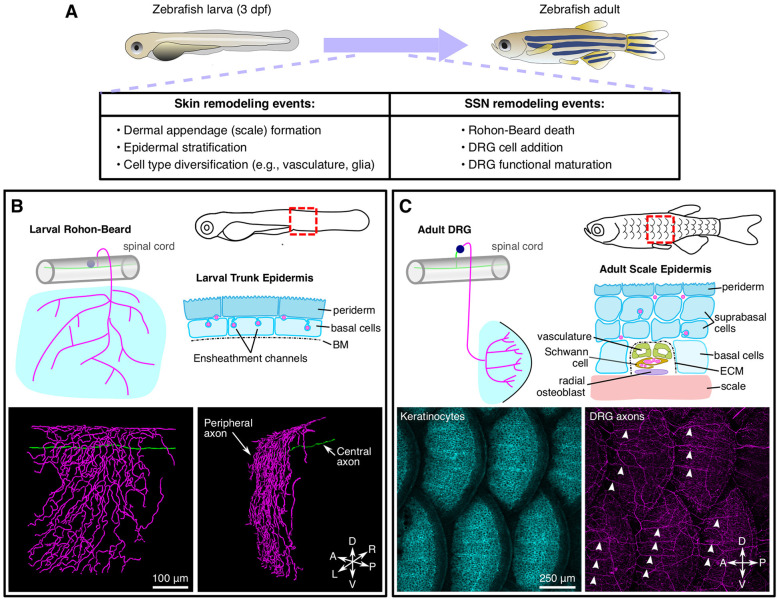
Anatomy and remodeling of zebrafish SSNs and skin. **(A)** Overview of changes to the skin and SSNs that occur during the larval-to-adult transition. **(B)** Anatomy of Rohon-Beard (RB) neurons in larval zebrafish. Top, illustration of the typical morphology of larval RB neurons along the trunk (left) and a transverse section through the epidermis (right). Bottom, partial reconstruction of a larval RB. Note that the peripheral axon predominantly arborizes along the dorsal-ventral (DV) axis. **(C)** Anatomy of dorsal root ganglion (DRG) neurons in adult zebrafish. Top, illustration of the typical morphology of DRG neurons innervating the adult scale epidermis along the trunk (left) and a transverse cross section through the epidermis surrounding a radial DRG axon bundle (right). Bottom, maximum intensity projections showing keratinocytes [labeled by *Tg(krt4:GFP)*] and DRG peripheral axons [labeled by *Tg(p2rx3a:lexa;lexaop:mCherry)*]. Note that the radial nerve bundles (arrowheads) orient anterior-posterior (AP) along the scale surface. Micrographs in **(C)** adapted from Rasmussen et al. (2018), with permission from Elsevier.

**Figure 4:**
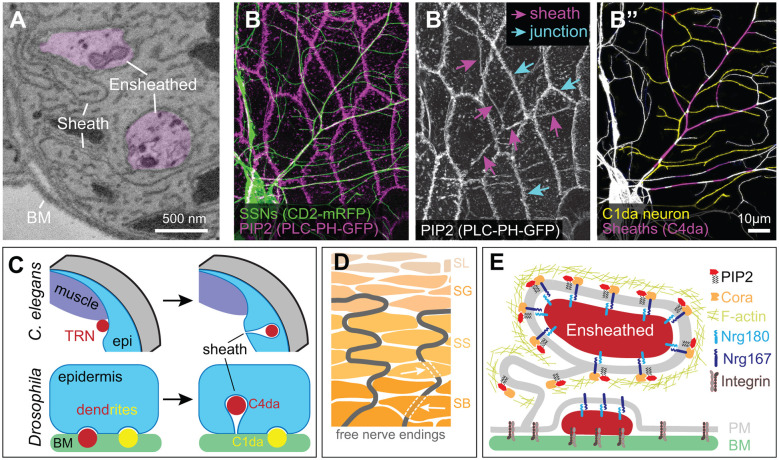
Ensheathment of SSN neurites by epidermal cells. **(A)** Serial block face scanning electron microscopy micrograph depicting ensheathed (purple) dendrites innervating the *Drosophila* larval epidermis. **(B)** Confocal micrograph depicting epidermal ensheathment of *Drosophila* SSNs. In this image, SSNs are labeled with a membrane-targeted fluorescent protein (green) and epidermal PIP_2_-positive lipid microdomains are labeled by PLCδ-PH-GFP (magenta), which accumulate at epidermal cell-cell junctions (cyan arrows) and at epidermal sheaths (magenta arrows) **(B’)**. **(B”)** Ensheathed and unensheathed dendrites often occupy overlapping lateral domains, and this is illustrated by the ensheathment of C4da dendrites (magenta) at sites of overlap with C1da dendrites (pseudo-colored yellow). **(C)** Developmental sheath assembly in *C. elegans* and *Drosophila*. TRN axons (ALM, PLM) are situated adjacent to dorsal muscle at the L1 stage, but following epidermal rearrangement in the L4 stage, the skin ensheaths TRN axons. *Drosophila* SSN dendrites grow on the basal surface of epidermal cells during embryonic and early larval development. Dendrites from a subset of these neurons (primarily dendrites of C4da neurons, indicated in red) induce membrane invagination and epidermal sheath assembly in third instar larvae. By contrast, unensheathed dendrites (yellow) remain at the basal epidermal surface. **(D)** Free nerve endings in human skin form cytoplasmic tunnels oriented perpendicular to the skin surface (indicated by white arrows) in keratinocytes within the stratum basale (SB) and stratum spinosum (SS) layers before arborizing within the stratum granulosum (SG; Talagas et al., 2020b). **(E)** Molecular components of *Drosophila* epidermal sheaths. Image credits: images in **(A–C)** are adapted from Jiang et al. (2019) under the Creative Commons License.

The larval epidermis derives from ~2,000 blastodermal precursors (Lohs-Schardin et al., 1979) that give rise to ~1,000 terminally differentiated epidermal cells per segment (Jiang et al., 2014). These cells form a monolayer of polarized epithelial cells with a basal lamina (Prokop et al., 1998), an apical cuticle, and lateral junctional domains (Figure 2A). Notably, the cell divisions that populate the larval epidermis occur during embryogenesis, and larval skin grows by epidermal cell expansion rather than cell division. As a result, spatial relationships between epidermal cells and SSNs can be traced throughout larval development (Parrish et al., 2009; Jiang et al., 2014).

Each larval segment contains >10 different epidermal cell types, transcriptionally specified on the basis of their position along the anterior-posterior (AP) axis within each parasegment (DiNardo et al., 1994). One manifestation of these different cellular identities is the stereotyped segmentally repeating pattern of cuticular protrusions on the apical surface of the epidermis, referred to as denticles (Lohs-Schardin et al., 1979; Bejsovec and Wieschaus, 1993). Although the positional information encoded in these different cell types likely influences dendrite morphogenesis, morphogenetic differences of these different epidermal cell types are not readily apparent on their basal surface. Instead, the monolayer of epidermal cells is composed primarily of tiled epidermal cells with a collagen-rich BM (Borchiellini et al., 1996), interspersed with apodemes, which serve as sites of body wall muscle attachment. In addition to these two prominent cell types, the epidermal layer contains stem cells (histoblasts) that repopulate the epidermis after metamorphosis, the resident neurons innervating the epidermis, and their accessory cells. Underneath the BM, the larval skin contains a number of non-epidermal cells that likely contribute to SSN development including specialized secretory cells (oenocytes), hemocytes (*Drosophila* blood cells), and muscle.

### D. rerio

Like *C. elegans* and *Drosophila*, *D. rerio* (zebrafish) have distinct experimental advantages for the analysis of SSN/skin interactions. Zebrafish are amenable to forward and reverse genetic screens; reverse genetic manipulation is particularly attractive since the large, externally fertilized eggs are easy to inject with antisense morpholino oligonucleotides or CRISPR-Cas9 ribonucleoprotein complexes. The small size of the larvae and automated behavioral assays additionally make high-throughput chemical screens feasible (Curtright et al., 2015).

Relative to *C. elegans* and *Drosophila*, zebrafish have additional anatomical complexity of both SSNs and skin (Figure 3). Larval zebrafish possess two types of SSNs—trigeminal (TG) and Rohon-Beard (RB) neurons—that originate from the neural plate border in neurula stage embryos and innervate the epidermis. TG and RB neurons generally share gene expression signatures, genetic requirements, and functional properties. Anatomically, TG neurons form bilaterally symmetric ganglia immediately posterior to the eyes, whereas RB neurons form along the rostral-caudal axis of the spinal cord. Whereas invertebrate SSNs elaborate peripheral processes with dendritic characteristics (containing mixed microtubule polarity, satellite secretory machinery, etc.; reviewed in Rolls and Jegla, 2015), vertebrate SSNs extend a single process that bifurcates to form central and peripheral projections both with axonal characteristics (reviewed in Nascimento et al., 2018). Zebrafish TG and RB neurons project peripheral axons that travel a short distance (dozens of microns) before reaching the epidermis where they branch profusely (O’Brien et al., 2012; Figure 3B). TG peripheral axons innervate the epidermis of the head and anterior yolk, whereas RB peripheral axons innervate the epidermis of the posterior yolk, trunk, and larval fin fold (Sagasti et al., 2005). TG somata reside in the TG ganglia and their central axons project caudally before entering the hindbrain. By contrast, RB somata localize in the dorsal spinal cord and their central axons run rostrally and caudally within the spinal cord. The central axons of both populations form connections with higher–order CNS neurons (Kimmel et al., 1990a; Palanca et al., 2013). TG and RB populations can be further divided into subtypes based on molecular markers (Slatter et al., 2005; Pan et al., 2012; Gau et al., 2013, 2017; Palanca et al., 2013), although the precise number and functional properties of the subtypes remain incompletely characterized.

A third population of SSNs, DRG neurons, develop at a later stage compared to RB and TG neurons. DRG neurons originate from the neural crest and form segmentally repeating ganglia adjacent to the spinal cord containing the DRG somata. In larvae, DRG peripheral axons appear to navigate to the periphery between muscle quadrants (Reyes et al., 2004), although this organization has not been extensively analyzed. DRG central axons penetrate the spinal cord through the dorsal root entry zone (Smith et al., 2017; Nichols and Smith, 2019) and elaborate projections within the spinal cord. Intriguingly, at these larval stages DRG neurons do not express many of the sensory receptors that function in TG and RB neurons (e.g., Piezo2b, Trpv1, Trpa1b; Prober et al., 2008; Pan et al., 2012; Faucherre et al., 2013; Gau et al., 2013; Esancy et al., 2018), suggesting that DRG neurons mature at a later stage and/or have distinct functional properties in larvae.

In contrast to the monolayered epidermal structures of worms and flies, zebrafish larvae have a bilayered epidermis. The (outer) periderm layer is derived from the enveloping layer (Kimmel et al., 1990b), which forms an early barrier between embryonic cells and the aquatic environment (Kiener et al., 2008). By contrast, the (inner) basal layer is derived from ventral ectoderm and forms later in development (Bakkers et al., 2002). Given these disparate origins, it is not surprising that periderm and basal cells have distinct genetic requirements. For example, specification of the basal, but not periderm, layer requires expression of an isoform of tumor protein 63 (Tp63) lacking N-terminal sequences (ΔNp63; Bakkers et al., 2002; Lee and Kimelman, 2002). The two epidermal layers also have distinct morphological features: the apical surface of the periderm is covered by labyrinthine actin-based structures known as microridges (Lam et al., 2015), whereas the cuboidal basal layer is attached to an underlying BM *via* hemidesmosomes (Sonawane et al., 2005). TG and RB axonal processes penetrate through the ECM and arborize directly between the periderm and basal layers (O’Brien et al., 2012). At these early larval stages, the epidermis contains additional cell types such as ionocytes, which regulate zebrafish skin physiology (Chang and Hwang, 2011).

During post-larval stages, both the skin and somatosensory system significantly remodel (Figure 3A). As the skin expands during post-larval growth, it undergoes two major morphogenetic changes. First, the bilayered epidermis begins to stratify through the proliferation of the Tp63-positive basal cell layer (Guzman et al., 2013; Rangel-Huerta et al., 2021). As the skin stratifies, the larval periderm is sloughed off and replaced by basal cell derivatives (Lee et al., 2014). Second, elements of the dermal skeleton, including scales and fin rays, form and reshape the overlying epidermis (Le Guellec et al., 2004; Parichy et al., 2009). In addition to these changes in the skin, the zebrafish somatosensory system also remodels during post-larval growth with DRG neurons eventually replacing RB neurons along the trunk. Early studies suggested that RB neurons were largely eliminated *via* apoptosis as early as 3 dpf (days post fertilization; Williams et al., 2000; Cole and Ross, 2001; Svoboda et al., 2001). However, several studies found that at least some RB neurons survive past 5 dpf (Reyes et al., 2004; Slatter et al., 2005; Palanca et al., 2013). Recent longitudinal tracking of RB neurons for ~2 weeks demonstrated that surviving RB neurons undergo morphological changes (Williams and Ribera, 2020), perhaps indicating why early studies based on histology concluded that they disappeared. How does the elimination of RB neurons correspond to when DRG neurons innervate the trunk? Imaging of a DRG-specific reporter and analysis of mutants lacking DRG, but not RB, neurons, suggested that the transition in SSNs may occur during scale morphogenesis (Rasmussen et al., 2018), much later than originally proposed. Strategies that allow unambiguous long-term tracking of RB neurons would help clarify when exactly the switch in skin innervation occurs and how this transition corresponds to the events of skin organogenesis.

## Control of Innervation Patterns within The Epidermis by Secreted Cues

Vertebrate embryology studies dating back over a century provided some of the first evidence that epidermal cues govern SSN arbor growth. In Harrison’s seminal studies, epidermal tissue promoted sprouting of DRG neurons in spinal cord explants (Harrison, 1910). By contrast, amputation of limb buds demonstrated that SSN innervation requires peripheral tissues (Shorey, 1909). Forty years after Shorey’s studies, Viktor Hamburger and Rita Levi-Montalcini demonstrated that peripheral tissues supply pro-survival cues to SSNs (Hamburger and Levi-Montalcini, 1949), providing the conceptual framework for the neurotrophin hypothesis and discovery of the first neurotrophin, nerve growth factor (NGF). Neurotrophins are perhaps the most widely studied family of extrinsic factors regulating SSN development, with innervation patterns governed by skin expression of neurotrophins and SSN expression of the cognate receptor. The many roles of neurotrophins in vertebrate SSN growth, survival, maintenance, and synapse formation have been extensively reviewed (Harrington and Ginty, 2013). Here, we focus on other classes of epidermal signals that shape SSN arbors.

Chemoattractants and repellents classically studied for roles in midline axon guidance comprise one major group of epidermal guidance cues that orient SSN neurite position in the periphery; gradients of growth factors similarly contribute to innervation patterns. Studies of axon targeting provided early indications that epidermal sources of secreted guidance cues shape SSN innervation. For example, circumferential migration of pioneer axons in *C. elegans* relies on epidermal UNC-5/Netrin (Hedgecock et al., 1990), graded epidermal Slit expression guides longitudinal axon outgrowth in *C. elegans* (Hao et al., 2001), and embryonic PNS axon pathfinding to the CNS in *Drosophila* (Parsons et al., 2003). Epidermal expression patterns for these molecules have not been systematically examined, but genetic studies demonstrate key principles of how they shape SSN peripheral arbors. First, these guidance molecules are expressed in discrete spatiotemporal epidermal domains, facilitating regional control of skin innervation. Second, a given guidance cue can exert distinct functions in different contexts. Third, these molecules act in combination: a given neuron can respond to multiple cues, sometimes at different stages. Fourth, individual neurons can respond to different guidance cues within the same territory, with a given guidance molecule acting on a subset of SSNs that encounter it. This selectivity is presumably achieved *via* cell type-specific expression of guidance receptors, facilitating neuron type-specific patterns of skin innervation.

### Regional Control of Skin Innervation

Somata of invertebrate SSNs are located in the periphery, where localized epidermal guidance cues orient process outgrowth. In *C. elegans*, ALM and PLM neurons extend a long anterior-directed process that branches and forms synapses, and a short posterior-directed process that neither branches nor forms synapses (Figure 1A). Both ALM and PLM rely on extrinsic Wnt signals to orient neurite outgrowth, but with distinct receptor-ligand pairs. Mutations in *lin-44* and *lin-17*, which encode a Wnt ligand and its receptor Frizzled, respectively, invert PLM neurite outgrowth (Hilliard and Bargmann, 2006; Prasad and Clark, 2006). LIN-17/Frizzled functions in PLM neurons, where it is targeted to posterior neurites, and expression of LIN-44/Wnt in posterior epidermal cells acts to polarize LIN-17 distribution in PLM (Herman et al., 1995; Hilliard and Bargmann, 2006). However, ectopic LIN-44 expression in anterior hypodermal domains partially rescued *lin-44* mutant PLM polarity defects, suggesting that additional positional cues may aid in orienting PLM neurite outgrowth (Hilliard and Bargmann, 2006). The situation with ALM is more complex: five Wnt ligands contribute to ALM outgrowth (Prasad and Clark, 2006; Chien et al., 2015), one of which (LIN-44) acts in an inhibitory fashion (Fleming et al., 2010). ALM utilizes distinct Wnt receptors from PLM, with the receptor MOM-5/Frizzled and kinase CAM-1/Ror required for Wnt-mediated ALM polarity. CAM-1/Ror provides an additional level of control, as CAM-1 exhibits antagonistic non-autonomous functions, presumably to inhibit inappropriate polarization.

Local epidermal guidance molecules similarly direct polarized outgrowth of *Drosophila* SSN neurites. Chordotonal (cho) organs contain bipolar mechanosensory neurons that extend a single unbranched dendrite, and a subset of these neurons (v’ch1 and lch5) migrate along the epidermis to their final position, rotating during migration to orient dendrite outgrowth (Bier et al., 1990; Salzberg et al., 1994). v’ch1 and lch5 take different routes, migrating dorsally and ventrally, respectively, and utilize distinct targeting mechanisms. Epidermal Netrin guides v’ch1 neurons; mutations in *Netrin-A (NetA)* or *frazzled (fra)*, which encodes an attractive DCC (deleted in colorectal cancer) family Netrin receptor, prevent v’ch1 migration and randomize the direction of dendrite outgrowth (Mrkusich et al., 2010). A patch of epidermal cells express NetA at the v’ch1 migratory destination during embryogenesis (Mitchell et al., 1996; Mrkusich et al., 2010), and ectopic NetA expression in lateral epidermis mistargets v’ch1 neurons. In this context, Fra functions in accessory cap cells that extend processes and migrate dorsally towards the Netrin source, pulling v’ch1 neurons to their final destination. Amphid neurons in *C. elegans* utilize a similar morphogenetic mechanism; these neurons form a multicellular rosette with accessory cells, and the tip of the rosette is tethered by adhesive interactions to migratory epidermal cells and towed to the nose (Fan et al., 2019). By contrast, lch5 neurons require Robo receptors and Slit for their guidance (Parsons et al., 2003; Kraut and Zinn, 2004; Gonsior and Ismat, 2019). Although Slit sources that guide lch5 migration have not been defined, mesodermal cells and lateral epidermal cells express Slit at the time of lch5 migration (Parsons et al., 2003).

A related mechanism determines the afferent innervation pattern of a class of low threshold mechanoreceptors (Aδ-LTMRs) in mouse skin, in which a local secreted factor orients neurite positioning (Rutlin et al., 2014). Aδ-LTMR fibers innervate hair follicles in a polarized fashion that corresponds to their directional tuning of hair deflection, and this polarized innervation depends on the neurotrophin brain-derived neurotrophic factor (BDNF) from the hair follicle epithelium. Caudal hair follicle epithelial cells selectively express BDNF, and conditional BDNF knockout in hair follicles attenuates the polarization of Aδ-LTMR endings.

### One Guidance Cue, Multiple Functions

Studies of Robo receptor function in *Drosophila* SSNs illustrate the multifunctional role of some epidermal guidance molecules in SSN development. First, Robo-mediated cho axon guidance away from the periphery and cho migration/orientation in the epidermis likely depend on distinct sources of repulsive Slit signals. Da neuron dendrite patterning also involves Robo function at multiple morphogenetic steps, but with added complexity. Da neurons are clustered at regular segmental locations (Figure 2A), each containing multiple neuron classes whose dendrites intermingle (Grueber et al., 2002). Dorsal cluster da neurons initially target dendrites dorsally towards the midline (Gao et al., 1999; Sugimura et al., 2003), and mutations in *Slit*, *Robo1*, and to a lesser degree *Robo2* lead to exuberant dorsal dendrite elongation in C4da neurons (Dimitrova et al., 2008), suggestive of repellent activity from the dorsal midline. One plausible model is that Robo receptors accumulate on nascent dendrite tips in C4da neurons, as in lch5 neurons (Kraut and Zinn, 2004; Gonsior and Ismat, 2019), to prevent dendrite growth beyond receptive field boundaries. Although the relevant Slit sources have not been identified, myocardial cells near the dorsal midline (Qian et al., 2005) and muscle attachment sites at segment boundaries (Kramer et al., 2001) produce Slits. Of note, other da neurons exhibit similar dorsal-directed outgrowth and express Robo receptors, yet only C4da neurons require Robo for proper dorsal outgrowth; whether differential Robo trafficking, coreceptors, or downstream transduction machinery is responsible for these differences remains to be determined.

Following embryonic outgrowth, Robo promotes dendrite branching and branch dynamics in larval C4da neurons: *Robo1* mutant single neuron clones exhibit significantly fewer terminal dendrite branches, and *Robo1* overexpression drives terminal dendrite stabilization and elongation (Dimitrova et al., 2008). Slit ligands display branch-promoting activities in zebrafish and mouse TG peripheral axons (Yeo et al., 2004; Ma and Tessier-Lavigne, 2007), so the requirement for *Robo1* in C4da neurons could reflect another branch-promoting activity for Slit. Antibody staining suggests that larval da neurons express Slit, hence spatially and temporally distinct Slit sources may tune guidance and growth of the same dendrite arbor *via* repulsive and attractive mechanisms. Intriguingly, one protease that mediates N-terminal Slit cleavage and conversion to an attractive cue has been identified (Kellermeyer et al., 2020), so it seems plausible that different forms of Slit mediate these different functions. Alternatively, one or more of these Robo functions may include non-Slit ligands: Robo functions as a coreceptor for Wnts and Netrins (Zelina et al., 2014; Wang and Ding, 2018).

### One Neuron, Multiple Guidance Cues

During metamorphosis, C4da neurons prune their dendrite arbors (Kuo et al., 2005), and yet another set of guidance cues shape subsequent dendrite regrowth. As in larvae, adult C4da dendrites tile the dorsal and lateral body wall, with ddaC and v’ada dendrites filling adjacent domains in a non-overlapping fashion (Shimono et al., 2009). Similarly, ddaC dendrites constrain dorsal growth of v’ada arbors, but ventral-directed branches terminate before encountering contralateral v’ada dendrites at the midline (Yasunaga et al., 2015). Thus, limited growth capacity or inhibitory signals constrain the ventral extent of v’ada arbors. Consistent with the latter, v’ada dendrites terminate at sternites, and genetic removal of sternites led to the ventral expansion of v’ada dendrite arbors. A genetic screen revealed that *Wnt5* mutation similarly drove the ventral expansion of v’ada dendrites, and indeed sternites express Wnt5, which concentrates at the dendrite-sternite boundary. Within neurons, Wnt5 repulsive cues are transduced by the Ryk receptor tyrosine kinases Derailed (Drl) and Drl2, which signal through the Rho guanine nucleotide exchange factor (GEF) Trio and Rho1 to locally destabilize the actin cytoskeleton and prevent dendrite extension beyond the Wnt5 source (Yasunaga et al., 2015).

Wnt signaling confers positional information for peripheral branch placement in *C. elegans* as well. The anterior-directed PLM process branches once at a stereotyped time and location, with F-actin coalescing into a patch at the future branch site prior to branching (Chen et al., 2017). Secreted Wnts signal through the receptor MIG-1/Frizzled to antagonize F-actin assembly, restricting branching at inappropriate locations along the AP axis. This Wnt signaling functions in concert with attractive Netrin cues that direct the PLM branch ventrally, and although the source for this Netrin cue has not been defined, epidermis-derived heparan sulfate proteoglycans (HSPGs) may contribute to the signaling (see below). Finally, as described above, oriented PLM neurite outgrowth relies on a distinct combination of guidance receptor and epidermal guidance cues.

### Combinatorial Functions of Guidance Signals

Studies of *Drosophila* Netrin (NetB) and Wnt (Wingless) ligands illustrate two additional principles of peripheral guidance by secreted cues. First, extrinsic guidance signals can work in concert with intrinsic mechanisms for neurite spacing to fine-tune peripheral arborization patterns. Self-avoidance signaling mediated by the *Drosophila* homophilic adhesion molecule Dscam1 promotes sister dendrite spacing in da neurons (reviewed in Zipursky and Grueber, 2013), but the loss of *Dscam1* drives inappropriate C3da dendrite targeting towards cho organs (Matthews and Grueber, 2011). This targeting is a consequence of Fra-mediated attraction towards NetB, which is expressed by accessory cells of the lch5 cho organ. Second, concentration gradients of secreted cues encode positional information in the epidermis. The *Drosophila* C1da neuron ddaE has a posterior facing comb-like dendritic arbor that develops in a highly stereotyped sequence (Sugimura et al., 2003; Parrish et al., 2006). Prior to ddaE dendrite outgrowth, the posterior epidermal cells within each abdominal segment exhibit graded expression of the Wnt ligand Wingless, with the most concentrated patch of Wingless dorsal to ddaE (Li et al., 2016). In this context, Wingless acts as a repellent for ddaE dendrite growth, with the trajectory of dorsally and ventrolaterally-directed branches shaped by the Wingless concentration gradient. Genetic epistasis analysis supports a cell-autonomous role for Frizzled receptors in transducing the Wingless signal in neurons, in part through controlling activity of the small GTPase Rac. However, in the absence of Wingless signaling, ddaE branches still largely orient along the AP axis suggesting that additional guidance cues orient these branches (see below).

## Epidermal Growth-Promoting Signals

SSNs have extreme growth requirements, in many cases projecting axons over vast distances and elaborating expansive peripheral arbors. Furthermore, peripheral arbors of SSNs exhibit cell type-specific diversity in their size, morphology, and developmental timing. While many of these features are a product of cell type-specific transcriptional (Dong et al., 2015) and translational programs (Lin et al., 2015), the epidermis controls important aspects of SSN growth. Different types of SSNs coexist in the periphery and experience the same extracellular cues, so how do epidermal cues tune SSN growth in a cell type-specific fashion? An emerging mechanism for this SSN type-specific growth control is signaling through receptors whose expression or signaling capacity is limited to specific types of SSNs.

### Permissive Signals for Neurite Growth

Genetic studies in *Drosophila* revealed that epidermis-derived HSPGs control skin innervation in a cell type-specific fashion. Genetic manipulations blocking HS chain biogenesis in epidermal cells severely disrupted dendrite growth in C4da neurons, but not other da neurons (Poe et al., 2017). Likewise, simultaneously knocking down epidermal expression of the HSPGs Dally (a GPI-anchored glypican) and Syndecan interfered with C4da dendrite growth. HSPGs are ECM components that signal locally through interactions with cell surface receptors and influence long–range signaling through effects on morphogen diffusion. HSPG control of C4da dendrite growth was local: blocking HSPG biogenesis in patches of epidermal cells disrupted dendrite growth over that patch but not over neighboring cells. Intriguingly, HSPGs do not appear to disrupt dendrite growth over apodemes; this may reflect spatial differences in growth-promoting cues within the epidermis. How do HSPGs regulate dendrite innervation? Time-lapse imaging revealed that HSPGs locally stabilize nascent dendrites, likely a consequence of dendritic microtubule stabilization. In the context of synapse development, syndecans and glypicans signal through receptor protein tyrosine phosphatases (RPTPs; Condomitti and de Wit, 2018), however, known RPTPs that bind Dally and Syndecan were dispensable for C4da dendrite growth (Poe et al., 2017). Hence, neuronal receptors responsive to *Drosophila* epidermal HSPGs remain to be identified. Finally, HSPGs may also regulate epidermal signal transduction that supports SSN neurite growth, as a recent study identified epidermal requirements for the RPTP CLR-1 in PVD dendrite growth (Liu et al., 2016).

In contrast to the relatively simple organization of the invertebrate epidermis, vertebrate skin is composed of a multi-layer stratified epidermis, presenting vertebrate SSNs with additional targeting decisions, including navigation to the periphery and guidance to the proper cell layer. Epidermal lesion studies in chicks revealed that, in addition to survival cues required to maintain peripheral projections, embryonic epidermis provides signals that promote skin innervation (Martin et al., 1989). Genetic studies in zebrafish revealed that epidermal HSPGs comprise one of these cues (Wang et al., 2012). Although HSPGs are present throughout the embryo, they are enriched in the epidermis, particularly the BM where axons enter the skin, suggestive of a role in directing skin innervation. Indeed, inactivating leukocyte antigen-related (LAR) family RPTPs disrupted skin innervation by RB neurons, leading peripheral arbors to branch and arborize beneath the skin, whereas wild-type neurons branch only after entering the skin. More importantly, genetic mutations that perturbed HSPG synthesis prevented appropriate skin innervation by RB neurons, as did exogenous application of an enzyme (heparinase) that degrades HSPGs. The latter treatment was particularly illuminating as focal heparinase injection created small patches of HSPG-deficient skin, and RB axon innervation was locally reduced within these HSPG-deficient patches.

In addition to effects on local neurite-ECM interactions, epidermal HSPGs can exert effects at a distance to create permissive growth environments. For example, ventral axon guidance of *C. elegans* AVM mechanosensory neurons requires Netrin secreted from the ventral midline (Hedgecock et al., 1990), and recent studies suggest this UNC-6/Netrin signaling relies on the hypodermal expression of the HSPG LON-2 (Blanchette et al., 2015). First, *lon-2* mutants exhibit axon guidance defects that resemble *unc-6* mutants. Intriguingly, cultured cells secrete LON-2 and hypodermal expression of a secreted form of LON-2 fully rescues *lon-2* function in AVM axon guidance. The finding that secreted LON-2 associates with UNC-40/DCC-expressing cells *in vitro* supports a model that LON-2 directly or indirectly interacts with UNC-40/DCC to modulate Netrin signaling and defines a new mode of signaling for epidermal-derived HSPGs. Similarly, the HSPG UNC-52/Perlecan appears to control dendrite branching of PVD neurons through effects on the extracellular environment that influence UNC-40/DCC-Netrin signaling (Celestrin et al., 2018).

### Growth Control by Secreted Factors

*Drosophila* C4da neurons are space-filling neurons with the most expansive dendrite arbors among da neurons. Homotypic repulsive signals govern receptive field boundaries and dendrite spacing in these neurons, but the TGF-β ligand Maverick (Mav) controls the density of body wall innervation (Hoyer et al., 2018). Epidermal cells secrete Mav, and ectopic Mav expression in patches of epidermal cells leads to localized increases in innervation. Likewise, *mav* knockdown in small epidermal patches locally decreases the dendrite density, whereas knockdown in large patches broadly affects C4da dendrite branching, suggestive of local and long–range effects on dendrite growth. How does Mav control dendrite branching? Mav signals through the receptor tyrosine kinase Ret, and C4da neurons internalize Mav in a Ret-dependent manner. Mav exhibits limited diffusion, so following dendrite growth into territories containing Mav, extracellular Mav is depleted by internalization, constraining exuberant growth.

These studies raise several interesting questions. First, what is the nature of the signal transduction pathway by which Ret locally controls dendrite growth? Loss of *Ret* or *mav* enhances dendrite dynamics, suggesting that Ret-Mav signaling promotes dendrite stabilization (Hoyer et al., 2018). Further, *Ret* mutation leads to local F-actin accumulation in dynamic dendrite branches, and Ret functions together with Rac to mediate integrin-based ECM adhesion (see below; Soba et al., 2015), so it seems likely that Ret-Mav signaling likewise modulates dendritic cytoskeletal assembly. Second, what coreceptor contributes to Ret-dependent dendrite growth control? Ret signals together with a variety of membrane proteins including the GPI-linked family Ret coreceptors GFRα1–3 (Harrington and Ginty, 2013), ephrins (Bonanomi et al., 2012), and integrins (Soba et al., 2015), however, *Drosophila* lacks identifiable GFRα homologs, hence Ret-Mav signaling is likely GFRα-independent. The most plausible candidate is integrin given the requirement for Ret in integrin-mediated ECM adhesion in these dendrites (see below). Third, how are short- and long-range Ret signaling coordinated? Mav exerts short and long-range effects on C4da dendrite growth, yet Mav exhibits limited diffusion, so internalized Mav likely regulates growth throughout the arbor. In vertebrates, internalized Ret-GDNF complexes mediate long–range retrograde signaling from the periphery together with GFRα1–3 (Harrington and Ginty, 2013; Tuttle et al., 2019), but short–range signaling events have been less extensively characterized. *Drosophila* Ret contains a single isoform, so this system presents an opportunity to parse local and long–range Ret signaling functions without the additional complexity of Ret isoforms with different intracellular domains and trafficking properties (Tsui and Pierchala, 2010; Tuttle et al., 2019). Finally, does Ret similarly regulate SSN peripheral arbor growth together with TGF-β ligands in vertebrates? Approximately 60% of mammalian DRG neurons express Ret (Molliver et al., 1997), but studies of Ret control of epidermal innervation patterns have largely focused on GDNF signaling (Luo et al., 2007, 2009).

In addition to locally producing and secreting growth-promoting factors, epidermal cells coordinate diffusible cues provided from other sources; studies of *C. elegans* PVD dendrite morphogenesis illustrate this form of growth control. The secreted factor LECT-2 is a muscle-derived cue required for PVD higher-order dendrite branching (Díaz-Balzac et al., 2016; Zou et al., 2016). LECT-2 accumulates at sites of dendrite formation in the epidermis, where it interacts with SAX-7/L1CAM. Epidermal SAX-7/L1CAM functions as part of an intercellular multiprotein complex together with the neuronal leucine-rich repeat protein DMA-1 and the secreted protein MNR-1 that drives dendrite branching (see below), and epistasis analyses demonstrated that LECT-2 functions together with this complex (Díaz-Balzac et al., 2016; Zou et al., 2016). Indeed, immunoprecipitation and cell aggregation assays demonstrated that LECT-2 enhances DMA-1/MNR-1/SAX-7 complex formation (Zou et al., 2016), hence an epidermal receptor (SAX-7) cooperates with a long–range signal (LECT-2) to spatially pattern dendrite growth.

Finally, an underexplored question is the extent to which epidermal cues attenuate neurite growth and/or drive denervation of territories after peripheral arbors have been established. *Drosophila* epidermal cells constrain SSN dendrite growth to ensure the synchronous expansion of dendrites and epidermis during larval growth, and this growth inhibition relies on direct physical coupling of dendrites to epidermal cells (ensheathment, see below) as well as increased epidermis-ECM adhesion that is thought to reduce the permissivity of the ECM to dendrite growth (Parrish et al., 2009). One intriguing study from *C. elegans* provides an example of epidermal signals that drive dendrite regression. Dendrites of PVD neurons exhibit age-dependent degeneration; an epidermally expressed antimicrobial peptide (AMP), NLP-29, triggers this degeneration (Lezi et al., 2018). NLP-29 expression increases during aging or in response to infection and signals through a neuronal GPCR (NPR-12) to induce autophagy-mediated dendrite degeneration. Human skin cells express a diverse array of AMPs, some of which are induced by injury and deregulated in skin diseases (Kenshi and Gallo, 2008), so it will be intriguing to determine whether AMP-mediated autophagy drives neurite degeneration in human skin.

## Control of SSN Neurite Position by ECM Interactions

A complex repertoire of direct and indirect adhesive interactions between neurites, the ECM, and epidermal cells precisely position SSN neurites (Figure 4). Dendrites of *Drosophila* da neurons arborize in a mostly 2D space on the basal surface of epidermal cells, positioned by integrin attachments to the BM (Han et al., 2012; Kim et al., 2012) that is likewise tethered to the epidermis by epidermal integrins (Jiang et al., 2014). Dendrite attachment to the BM requires epidermis-derived laminins, and reducing expression of neuronal integrins or epidermal laminins causes dendrites to reorient in 3D space and become embedded inside epidermal cells (see below; Han et al., 2012; Kim et al., 2012). This axial repositioning results in out-of-plane dendrite-dendrite crossing events, demonstrating that ECM attachment is required to position neurites for avoidance signaling in *Drosophila*. Likewise, early anatomical studies of vertebrate SSNs noted that growing neurites readily cross below the innervated skin territories and only insert into the basal lamina when they reach non-innervated skin (Scott et al., 1981; Hayes and Roberts, 1983; Kitson and Roberts, 1983), suggesting that repulsive interactions between neurites requires ECM interactions and/or confinement to a 2D plane.

Dendrite positioning in da neurons is further regulated by additional factors that indirectly mediate ECM attachment. First, Ret functions together with integrins to regulate dendrite-ECM attachment (Soba et al., 2015). Ret additionally interacts with Rac, a GTPase, to regulate dendritic F-actin distribution and, similar to *Ret* mutants, Class IV da neurons with compromised Rac function are no longer confined to a 2D plane. It, therefore, appears that ECM adhesion by Ret/integrin regulates the actin cytoskeleton *via* Rac. Second, epidermally-secreted Semaphorin Sema-2b signals through neuronal Plexin B to activate the NDR family kinase Tricornered (Trc), which promotes ECM adhesion (Meltzer et al., 2016). Constitutive Trc activation suppresses ECM detachment defects of *Sema-2b* mutants, and Ndr kinases regulate integrin-based adhesions in multiple contexts: Trc inactivation in C4da results in ECM detachment defects (Han et al., 2012), one additional cell surface receptor (Raw) mediates integrin-based adhesions *via* Trc activation (Lee et al., 2015), and Ndr2 regulates integrin trafficking in hippocampal neurons (Rehberg et al., 2014). Given that PlexB receptors bind the beta-integrin Mys in dendrites, receptor activation of Trc may locally modulate integrin-based ECM contacts. Finally, indirect interactions with the ECM contribute to the positioning of sensory dendrites in both *Drosophila* and *C. elegans* (Jiang et al., 2014; Liang et al., 2015).

In contrast to these invertebrate systems, SSNs in vertebrates navigate through more complex skin, which presents several additional challenges. First, different neurons terminate in different dermal and epidermal layers, innervating particular territories within those layers. For example, peptidergic nociceptors terminate in the stratum spinosum (SS), whereas non-peptidergic nociceptors project through the SS and innervate the stratum granulosum (SG; Zylka et al., 2005). Differences in ECM composition across the skin likely direct these innervation patterns. Indeed, epidermal stem cells in mouse hair follicles deposit EGF-like domain multiple 6 (EGFL6) into the collar matrix, which ensheathes mechanosensory lanceolate complexes (Cheng et al., 2018), and axons of low threshold mechanoreceptors form stable integrin-based contacts with EGFL6. These attachments promote parallel patterning of axons and terminal Schwann cells in lanceolate complexes and also contribute to tactile acuity. Beyond this example, expression analyses of mammalian skin demonstrate that integrin expression varies across skin layers (Watt, 2002), that different dermal layers express different ECM components and contain different fibroblast populations (Rognoni and Watt, 2018), and that epidermal layers exhibit gradients of different proteoglycans (Sanderson et al., 1992), each of which likely shapes local innervation patterns. The latter is of note given the roles for proteoglycans as permissive cues for *Drosophila* C4da dendrites and the enrichment of zebrafish HSPGs beneath the basal cell layer which RB axons innervate (Wang et al., 2012).

As the vertebrate skin grows, not only does it stratify, but it also adds appendages, such as scales, feathers, and hair. As these appendages present significant local obstacles to epidermal innervation, how does the somatosensory system deal with this challenge? In adult zebrafish, scales are planar polarized, millimeter-sized bony plates that form a protective armor immediately below the epidermis. Analysis of the adult scale epidermis revealed that, in striking contrast to the larval trunk where RB peripheral axons arborize as individual fibers in a predominantly dorsal-to-ventral orientation, DRG peripheral axons entering the adult epidermis form bundles directed along the AP axis (Figures 3B,C; Rasmussen et al., 2018). In contrast to the “naked” larval peripheral axons, neural crest-derived Schwann cells ensheath these bundles, which run alongside vasculature (Figure 3C), similar to mammalian skin (Mukouyama et al., 2002). Developmental and genetic analysis demonstrated that axons and vascular patterning are mutually independent, but require scale osteoblast-mediated patterning. Early during scale morphogenesis, directed migration by a subset of scale osteoblasts creates radial tracts encased by a laminin-rich ECM along the scale surface. Axons, and later vasculature, then access the skin by migrating through these tracts. Blocking scale development resulted in significantly reduced axon and vascular density and a larval-like polarity of axon arborization. Mutants with reversed scale polarity also showed reversed axon polarity. Together, these results indicate that scales are necessary and sufficient for locally orienting axons in adult zebrafish. As described above, a conceptually similar polarized orientation of SSN axon fibers around mouse hair follicles requires epithelial expression of BDNF (Rutlin et al., 2014). In future studies, it will be interesting to assess whether SSN guidance along scales involves similar molecules or, rather, relies on an alternative mechanism such as haptotaxis.

## Direct Adhesive Interactions That Position Somatosensory Neurites

As with secreted factors, gradients of epidermal adhesion molecules influence peripheral arbor distribution, and studies of the Teneurin family homophilic adhesion molecule Ten-m demonstrate how a single adhesion molecule can dictate different arbor geometries in different neurons (Hattori et al., 2013). Ten-m expression is graded in the *Drosophila* larval epidermis, with expression high at the center and low at anterior and posterior boundaries of each segment, facilitating a gradient of homophilic Ten-m interactions that provides directional preference to dendrites. Indeed, high Ten-m expressing C1da dendrites strongly orient their dendrite branches along the epidermal Ten-m gradient, whereas low Ten-m expressing C4da dendrites exhibit directional preference only in the high-expressing epidermal Ten-m domain. Teneurins organize the cytoskeleton at synapses in part through contacts with alpha-spectrin, hence Ten-m adhesions could orient dendrite branches through direct control of cytoskeletal geometry (Mosca, 2015).

Do teneurins position SSN neurites in other systems? In *C. elegans*, neurons and epidermal cells express teneurin (TEN-1; Mörck et al., 2010), but *ten-1* mutants exhibit pleiotropic phenotypes, including defects in axon guidance along the flank and hypodermal cell migration (Drabikowski et al., 2005). Genetic interaction studies suggest that TEN-1 functions in BM assembly or maintenance in *C. elegans* (Topf and Drabikowski, 2019), and physical interactions between teneurins and other membrane receptors including integrins and latrophilin raise the possibility that vertebrate teneurins may mediate skin innervation by a variety of mechanisms. What other homophilic adhesion molecules might serve an analogous role in mammalian skin? Cadherins are appealing candidates given the many functions for cadherin-based adhesion in nervous system development and the graded expression of desmosomal cadherins in epidermal layers (Schäfer et al., 1994).

In *C. elegans*, dendrites of mechanosensory PVD neurons innervate the muscle-epidermis interface (Figures 1B,C), and control of PVD dendrite arbor geometry by interactions between neuronal DMA-1 (a leucine-rich repeat transmembrane protein) and the epidermal SAX-7/L1CAM and MNR-1/Menorin co-ligand complex provides the most extensively characterized paradigm for the spatial patterning of dendrite arbors by direct interactions with the hypodermis (reviewed in Richardson and Shen, 2019; Jin and Kim, 2020). In this system, neuronal DMA-1 interacts with hypodermal SAX-7/L1CAM (an immunoglobulin superfamily cell adhesion molecule) and MNR-1 to spatially pattern primary, secondary, and tertiary PVD dendrites (Dong et al., 2013; Salzberg et al., 2013). The patterned distribution of SAX-7 in regular hypodermal stripes positions terminal PVD dendrites, and depleting SAX-7 or altering its distribution leads to loss or mistargeting of terminal dendrites, respectively (Dong et al., 2013; Salzberg et al., 2013; Liang et al., 2015; Zhu et al., 2017). Exclusionary interactions with the muscle-derived HSPG UNC-52/Perlecan controls SAX-7 distribution in the epidermis (Liang et al., 2015). UNC-52 is a major component of the ECM that covers muscle, with UNC-52 tethered to muscle in a striped pattern by virtue of integrin-based contacts. UNC-52 regulates the position of hypodermal hemidesmosomes, which connect hypodermal cells to the ECM, and SAX-7 interdigitates between UNC-52 stripes, which in turn directs growth of PVD 4° branches by interaction with DMA-1 and MNR-1. Signaling downstream of DMA-1 involves two distinct transduction mechanisms that promote F-actin assembly: recruitment of the Rac GEF TIAM-1 through interactions with the DMA-1 intracellular domain, and indirect recruitment of the WAVE complex *via* DMA-1 interactions with the claudin HPO-30 (Zou et al., 2016; Tang et al., 2019).

L1CAM/Neuroglian (Nrg) likewise regulates dendrite positioning of *Drosophila* SSN dendrites, albeit by a slightly different mechanism. In flies, epidermal cells and SSNs express Nrg isoforms which differ in intracellular but not extracellular domains and are therefore capable of interacting (Yamamoto et al., 2006; Yang et al., 2019). A series of genetic manipulations of neuronal (Nrg180) or epidermal (Nrg167) isoforms demonstrated that dendrite spacing in the epidermis critically depends on the balance of Nrg dendrite-dendrite and dendrite-epidermis interactions (Yang et al., 2019). Epidermal Nrg167 expression promotes dendrite arborization, potentially through stabilization of dendritic Nrg180. Furthermore, reduced Nrg167 expression in the skin or increased Nrg180 expression in neurons led to inappropriate bundling of dendrites. Hence, Nrg167 appears to tether dendrites to epidermal cells, counteracting the bundling induced by Nrg180 homophilic interactions.

## Specialized Epidermal-SSN Interactions

Many types of cutaneous receptors form specialized terminal structures with epidermal components that contribute to somatosensation (reviewed in Owens and Lumpkin, 2014). For example, low threshold mechanoreceptor afferents form synapse-like contacts with Merkel cells (Mihara et al., 1979), which respond to mechanical stimuli and tune gentle touch responses (Maksimovic et al., 2014). Similarly, afferent interactions with radially packed Schwann cell-derived lamellar cells in Pacinian corpuscles facilitate high–frequency sensitivity (Loewenstein and Skalak, 1966). Less is known about the structural and functional coupling of keratinocytes to SSN free nerve endings that innervate the epidermis, but studies in worms, flies, and fish have identified the developmental origin and potential functions of epidermal sheaths that wrap these SSN neurites.

### Developmental Origins of Ensheathment

Anatomical studies dating back more than 50 years suggested that epidermal cells physically wrap portions of free nerve endings (Munger, 1965). However, the lack of suitable markers for labeling the neurons meant that ensheathed neurites (also referred to as “enclosed” neurites in some studies, e.g., Han et al., 2012; Kim et al., 2012) could not be unambiguously distinguished from other peripheral cell extensions, such as dendritic processes of Langerhans cells (Kruger et al., 1981). Serial-section electron microscopy studies provided one solution to this problem; following SSN axons as they exit Schwann cells and insert into keratinocytes established that SSN axons indeed insert into epidermal cells (Cauna, 1973, 1980). Ultrastructural studies of *C. elegans* TRNs revealed that the hypodermis wraps neurites of ALM and PLM (Chalfie and Sulston, 1981), and studies in *C. elegans* provided the first clues about the developmental origin of epidermal sheaths. In newly hatched larvae, TRN neurites are located adjacent to the muscle (Emtage et al., 2004). During larval growth, the hypodermis extends between the TRN neurite and muscle, displacing the neurite from its position adjacent to the muscle and ensheathing the neurite (Figure 4C). *Drosophila* and zebrafish epidermal cells similarly wrap SSN neurites, with sheaths forming by membrane invaginations that wrap membranes around the entire circumference of the sensory neurite (Han et al., 2012; O’Brien et al., 2012; Kim et al., 2012). The wrapping epidermal membranes are tightly apposed to one another and the ensheathed neurites, embedding the neurites in a mesaxon-like structure that can extend over lengths of several microns or more (O’Brien et al., 2012; Jiang et al., 2019) and encompass >30% of C4da dendrite arbors in *Drosophila* (Jiang et al., 2018).

How are sheaths formed? Studies in *Drosophila* and zebrafish defined an evolutionarily conserved pathway for this morphogenetic event (Jiang et al., 2019). The earliest discernable event in this pathway is the formation of phosphatidylinositol 4,5-bisphosphate (PIP_2_)-enriched microdomains on epidermal membranes adjacent to sensory neurites (Figure 4E). As epidermal membranes invaginate to ensheath neurites, these microdomains extend along the entire length of the sheath. PIP_2_ is a negatively charged phospholipid that recruits proteins to the plasma membrane (De Craene et al., 2017), and PIP_2_ enrichment at nascent sheaths is followed by recruitment of the GTPase Rho1 and filamentous actin (F-actin) to the cortex of the epidermal membrane surrounding the invaginating neurite (Jiang et al., 2019). Finally, junctional proteins are recruited to sheaths, where they may seal sheaths and limit sheath permeability (Kim et al., 2012; Jiang et al., 2019). The nature of these autotypic junctions is not fully defined, but zebrafish sheaths contain both adherens junctions and desmosomes (Jiang et al., 2019), whereas *Drosophila* sheaths contain adherens junction (E-cadherin, β-catenin/armadillo) and numerous septate junction proteins (Discs large, Coracle/Band4.1, Nrg/L1CAM, Neurexin-IV, and Scribble, among others; Kim et al., 2012; Tenenbaum et al., 2017; Jiang et al., 2019; Yang et al., 2019). Although the molecular basis for the recruitment of these junctional proteins remains to be determined, studies in *Drosophila* suggest one plausible mechanism. Cora, the sole *Drosophila* erythrocyte membrane protein band 4.1 (EPB41) family member, is required for sheath formation, and EPB41 proteins function as interaction hubs that organize specialized plasma membrane domains (reviewed in Baines et al., 2014). Within the nervous system, the EPB41 family protein EPB41L2/4.1G is concentrated at membranes of Schwann cells (Ohno et al., 2006) and plays essential roles in Schwann cell ensheathment, in part through organizing glial transmembrane proteins (Ivanovic et al., 2012; Terada et al., 2019). Similarly, Cora organizes septate junctions (SJs) *via* interactions with Neurexin-IV and Nrg/L1CAM (Lamb et al., 1998; Ward et al., 1998) and is required for accumulation of Nrg at sheaths (Yang et al., 2019). Epidermal sheaths are enriched in PIP_2_, purified recombinant versions of the Cora FERM domain directly bind PIP_2_ (Nunomura et al., 2014), and PIP_2_ binding modulates EPB41 family binding specificity for membrane proteins (An et al., 2006). Hence, PIP_2_ accumulation may drive epidermal sheath maturation *via* the recruitment of Cora/EPB41.

The deep conservation of the pathway for sheath formation suggests that similar events likely govern sheath formation in mammals. However, sheaths may form by alternative pathways as well. Epidermally-embedded dendrites lacking identifiable sheath structures have been described in *Drosophila* (Han et al., 2012). Although these structures may represent instances of sheath loss, time-lapse imaging demonstrates that sheaths are remarkably stable structures (Jiang et al., 2019). Furthermore, the spatial distribution of these structures, which occur at epidermal intercellular junctions, is distinct from the majority of ensheathed dendrites, which occur on the basal face of epidermal cells. Some features of *C. elegans* sheaths likewise appear to be unique. First, *C. elegans* sheaths form by extending hypodermal cell membranes around target neurites rather than membrane invagination. Following hypodermal wrapping of TRN neurites, hemidesmosome structures form, anchoring neurite attachment to the hypodermis (Vogel and Hedgecock, 2001). Second, a specialized ECM surrounds ensheathed TRN neurites (Emtage et al., 2004); a specialized sheath ECM has yet to be identified in fish or flies.

### Sheath Organization

Several key principles governing sheath distribution have emerged. First, SSN ablation prevents sheath formation in *Drosophila* and zebrafish alike (Jiang et al., 2019) and sheath structures appear only at sites of neurite contact, suggesting that neurons initiate the process. Second, although epidermal cells ensheath different classes of SSNs to different degrees in both *Drosophila* and zebrafish (Jiang et al., 2019), the epidermal sheaths that wrap different SSN types appear structurally similar. Ablation studies have not revealed competitive interactions between neurons for sheaths, suggesting that competition for limited epidermal occupancy does not determine ensheathment levels. Thus, the levels of sheath-inducing signals(s) expressed by a particular SSN type likely determines the extent of ensheathment. Third, sheath formation is temporally regulated. In flies, worms, and fish SSNs innervate the epidermis hours or days prior to sheath formation (Emtage et al., 2004; O’Brien et al., 2012; Jiang et al., 2014). Fourth, sheaths generally contain only a single neurite (Jiang et al., 2019; Talagas et al., 2020b), possibly the consequence of neurite-neurite avoidance signals that limit neurite coincidence at sites of sheath formation (Yang et al., 2019). Fifth, different epidermal cell types have different capacities for ensheathment, and this is especially true in animals with a multilayered epidermis. In *Drosophila* larvae, most epidermal cells appear capable of forming sheaths with the notable exception of apodemes (Jiang et al., 2019). In the bilayered zebrafish larval epidermis, sensory axons innervate the region between the periderm and basal cell layer but only basal cells ensheath axons (O’Brien et al., 2012); similarly, structures resembling epidermal sheaths are apparent in the outer but not inner layers of the stratified human epidermis (Figure 4D; Talagas et al., 2020b). Recent studies characterizing transcriptional differences between these different epidermal cell types may facilitate identification of ensheathment machinery (Cheng et al., 2018; Cokus et al., 2019).

What are the identities of signals that drive sheath formation? First, integrins function cell-autonomously in SSNs to limit ensheathment: integrin knockdown enhances ensheathment of all SSNs, including normally unensheathed neurons (Han et al., 2012; Kim et al., 2012). Likewise, attenuating epidermal laminin production potentiates ensheathment (Han et al., 2012), suggestive of a dynamic interplay between adhesive interactions that drive ensheathment and ECM interactions that limit ensheathment. Dendrite-epidermis tethering mediated by Nrg/L1CAM potentiates epidermal SSN ensheathment (Yang et al., 2019). However, ensheathment is still observed in the absence of Nrg167 expression, Nrg167 mediates epidermal attachment of ensheathed and unensheathed dendrites alike, and Nrg180 levels do not covary with the level of ensheathment in different SSNs. Hence, additional signals likely dictate patterns of sheath formation.

### Functions of Epidermal Sheaths

Epidermal sheaths serve a variety of functions in SSN morphogenesis. First, epidermal ensheathment facilitates the coexistence of different SSN arbors in *Drosophila*. Most da neuron dendrites occupy a 2D territory on the basal surface of epidermal cells (Han et al., 2012; Kim et al., 2012; Lee et al., 2015), but ensheathed portions of arbors shift apically inside the epidermal monolayer, allowing other da neurons to innervate unoccupied basal space and “share” territory (Figures 4A,B,E; Tenenbaum et al., 2017).

Second, epidermal sheaths regulate SSN branching and structural plasticity (Jiang et al., 2014; Lee et al., 2015; Tenenbaum et al., 2017). After establishing complete body wall coverage, C4da dendrite structural plasticity is progressively restricted (Parrish et al., 2009), with epidermis and C4da arbors expanding in synchrony. In *Drosophila* larvae, the epidermally-expressed microRNA *bantam (ban)*, controls this developmental restriction of C4da plasticity (Parrish et al., 2009). Loss of *ban* function completely blocks epidermal ensheathment and causes dendrites to branch exuberantly. *ban* regulates ensheathment in part by increasing epidermal integrin expression and hence promoting epidermis-ECM interactions (Jiang et al., 2014). A similar mechanism may regulate permissivity to neurite growth in mammalian skin given the regional differences in integrin expression (Watt, 2002). *Ban* may additionally regulate the competence of epidermal cells to ensheath SSNs as *ban* expression precedes ensheathment and accelerating the timing of *ban* expression leads to precocious ensheathment and epidermal plasma membrane invagination (Parrish et al., 2009; Jiang et al., 2014). As with *ban* mutants, knockdown of epidermal factors required for sheath formation including Cora/Band 4.1 leads to changes in dendrite branch number and dynamics (Tenenbaum et al., 2017; Jiang et al., 2019). Finally, within the dendritic arbor of a single neuron, unensheathed dendrites exhibited enhanced dynamics and were less persistent than ensheathed dendrites. Therefore, epidermal sheaths promote dendrite stabilization and constrain dendrite growth.

Epidermal sheaths likewise promote long-term TRN axon maintenance in *C. elegans*. Longitudinal imaging demonstrated that mutations preventing TRN ensheathment caused blebbing and degeneration of adult TRN neurites (Pan et al., 2011). What is the source of these maintenance defects? One plausible explanation is that sheaths protect TRN neurites from mechanical damage. Indeed, a genetic screen for TRN stabilization factors identified requirements for epidermal UNC-70/β-spectrin and the Rab GTPase RAB-35 in sheath formation and protection of TRNs from mechanical damage (Coakley et al., 2020). UNC-70/β-spectrin and a RAB-35 GAP, TBC-10, accumulate along TRN sheath furrows. Inactivation of either *unc-70* or *tbc-10* led to deficits in TRN ensheathment, loss of hemidesmosome structures, which resist mechanical stress (Zhang et al., 2011), and TRN degeneration at sites of sheath loss (Coakley et al., 2020). Mutations that paralyzed *C. elegans* suppress these phenotypes, strongly suggesting that mechanical strain associated with locomotion drives hemidesmosome loss and axon fragmentation in *unc-70* and *tbc-10* mutants.

Finally, epidermal sheaths modulate SSN function in certain contexts. Blocking epidermal sheath assembly or maturation in *Drosophila* attenuates responses to noxious mechanical stimuli (Jiang et al., 2019). By contrast, epidermal wrapping of touch cells in *C. elegans* does not affect touch sensitivity (Chen and Chalfie, 2014), suggesting that structurally distinct sheaths can serve different functions. Keratinocytes release compounds that can modulate SSN function (Woolf et al., 1997; Koizumi et al., 2004; Moehring et al., 2018), hence it seems plausible that epidermal sheaths could function as release sites that functionally couple sheath-forming epidermal cells and SSNs. Such a scenario would be reminiscent of Merkel cell communication with SSNs (Mihara et al., 1979; Maksimovic et al., 2013, 2014; Woo et al., 2014; Hoffman et al., 2018). While some studies have suggested that keratinocytes express presynaptic release machinery that may be involved in neurotransmitter release (Talagas et al., 2020a), these studies are currently limited to *in vitro* co-culture experiments of DRG neurons and keratinocytes, so whether such contacts form *in vivo* remains to be determined. Some of the proteins that localize to epidermal sheaths, including Cora/Band 4.1 and Nrg/L1CAM, play established roles in synaptic organization, so it will be intriguing to see whether sheaths function as scaffolds for the release of signaling molecules that modulate SSN activity.

## Epidermal Pruning of SSNs

Both developmental remodeling and damage-induced degeneration of neurites require nearby phagocytes to aid in the pruning or removal of debris. On a tissue level, pruning or timely removal of debris after neurite degeneration is of paramount importance to reduce inflammation caused by lingering cell debris and facilitate possible reinnervation of target sites (reviewed in Coleman and Höke, 2020). This is a particular challenge for the skin given the enormous size, density, and complexity of peripheral cutaneous neurites, where a single ending in the mouse skin can reach ~1 meter in length (Wu et al., 2012).

**Figure 5:**
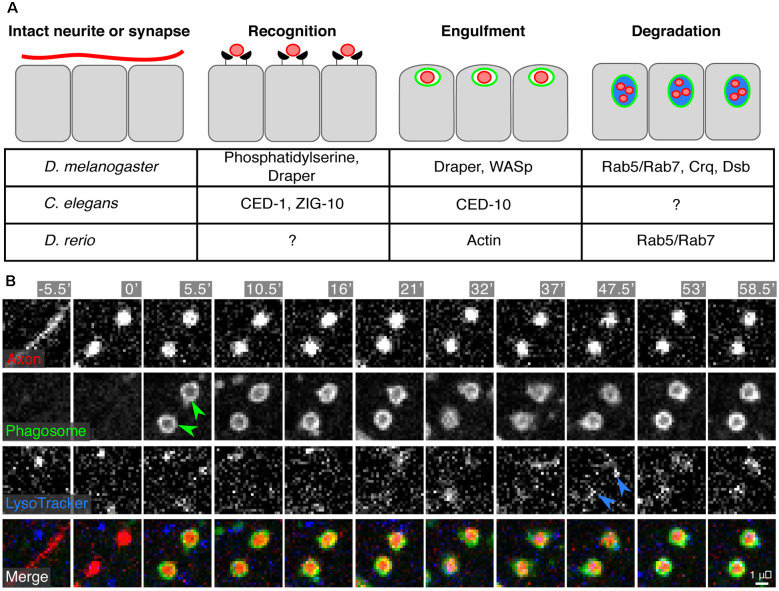
Engulfment of synapses or neurite debris by epidermal cells. **(A)** A synapse to be eliminated or a damaged neurite undergoes recognition, engulfment, and degradation by the surrounding epidermal cells. The currently identified molecular machinery for these processes is listed in the table. **(B)** Time-lapse microscopy of engulfment and degradation of SSN axon debris by epidermal cells following axotomy in larval zebrafish. Green arrowheads indicate engulfment and internalization of axon debris [labeled by *Tg(isl1[ss]:lexa;lexaop:tdTomato)*] into epidermal phagosomes [labeled by *TgBAC(p63:Gal4FF)*; *Tg(4×UAS:EGFP-2×FYVE)*]. Blue arrowheads indicate acidification of the phagosomal compartments as visualized by Lysotracker staining. Micrographs in **(B)** reprinted from Rasmussen et al. (2015) under the Creative Commons License.

In many other contexts, “professional” phagocytes mediate neuronal and neurite removal (e.g., microglia in the mammalian CNS). What are the cells that mediate engulfment and digestion of neurite debris in the skin? Surprisingly, neither macrophage-like hemocytes in *Drosophila* nor hematopoietic-derived cells in zebrafish play major roles in SSN debris engulfment in larval skin (Han et al., 2014; Rasmussen et al., 2015). These observations suggest that regulation of SSN homeostasis involves specialized aspects of the skin microenvironment and/or molecular mechanisms. Indeed, “non-professional” epithelial cells are the major phagocytic cell type for SSN debris in the epidermis of larval worms, flies, and zebrafish (Han et al., 2014; Rasmussen et al., 2015; Nichols et al., 2016). For example, in *Drosophila* larvae, epidermal cells mediate developmental pruning of C4da dendritic arbors (in particular, ddaC) and engulf dendrite debris generated *via* laser-induced damage (Han et al., 2014). Similarly, both layers of the larval zebrafish epidermis engulf axonal debris following laser-induced degeneration of SSN arbors (Figures 5A,B; Rasmussen et al., 2015). Interestingly, zebrafish epidermal cells can also engulf debris from other axonal or cell types, suggesting they are not tuned to recognize only SSN debris, and that studies of the mechanisms underlying neurite recognition and engulfment may yield broader insights into skin repair.

Epidermal and non-epidermal cells (such as glia) often rely on the same set of phagocytic machinery to recognize and engulf synapses, cell corpses, or debris (Figure 5A). In *Drosophila*, many studies have focused on Draper (*drpr*), an engulfment receptor, as an important component in both epidermal and non-epidermal phagocytic clearance of axon debris (Awasaki et al., 2006; MacDonald et al., 2006; Han et al., 2014). Indeed, pruning and engulfment of debris by fly epidermal cells depends on Draper (Han et al., 2014). Similarly, in worms, engulfment of axonal debris requires CED-1, a Draper homolog (Nichols et al., 2016). Additional work in *C. elegans* found that ZIG-10, a two-immunoglobulin domain transmembrane protein, regulates CED-1/Draper-mediated synapse clearance in the epidermis (Cherra and Jin, 2016). Similar to glia, epidermal cell engulfment involves reorganization of the actin cytoskeleton (*via* Rac1, CED-10, and WASp) downstream of engulfment receptors (Nichols et al., 2016). Lastly, exposure of phosphatidylserine by SSN neurites likely acts as a molecular cue for engulfment by epidermal cells, similar to pruning in the CNS and engulfment of apoptotic cells (Ravichandran, 2010; Sapar et al., 2018; Scott-Hewitt et al., 2020).

What is the fate of internalized neurite debris? Studies of the intracellular processing of debris in epidermal cells have revealed both old and new requirements. In zebrafish, debris acidification and processing requires the Rab5/Rab7 endosome maturation pathway classically used by professional phagocytes (Rasmussen et al., 2015). Epidermal phagosome maturation in *Drosophila* requires the CD36 family member Croquemort (crq; Han et al., 2014), which had been previously studied for its role in the clearance of apoptotic corpses in *Drosophila* (Franc et al., 1999). Furthermore, an RNAi screen identified *debris buster*, a novel component of the phagosome maturation pathway, highlighting the potential for studies of SSN degradation as a gene discovery tool for phagocytic regulators (Han et al., 2014).

Questions remain about epidermal involvement in neurite pruning and debris removal in adult animals, as well as whether these features are conserved in mammalian systems. Intriguingly, one recent study in mice found that epidermal SSN fibers often reside directly beneath keratinocyte tight junctions that form below the outer, cornified layer (Takahashi et al., 2019). In instances where new tight junctions are forming, epidermal keratinocytes can prune cutaneous neurites to keep them below the tight junctions. In a mouse model of epidermal barrier impairment (*Spade)* and in human skin samples from patients with atopic dermatitis (AD), epidermal fibers often penetrate through the tight junction barrier and avoid pruning by keratinocytes. These observations raise the interesting possibility that aspects of pathological itch in AD may be due to aberrant SSN pruning by epidermal cells. It is possible that similar mechanisms may be at play in other skin diseases, lending to their pathologies, but this requires more careful investigation.

## Future Prospects

Despite the recent progress, substantial questions remain to be answered about epidermal control of SSN innervation. One pressing question is the extent to which epidermal diversity contributes to innervation patterns. A necessary prerequisite to answering this question is a deeper sampling of epidermal cell types. Even within model systems, this question is understudied, hence leveraging positional information embedding in the *Drosophila* body plan and/or comparative analysis of zebrafish epidermal cells should provide insight into diverse epidermal functions in control of SSN innervation. How are epidermal signals integrated over space (long- and short–range) and time? *C. elegans* presents an appealing system to address these questions, given the morphological stereotypy and limited cellular diversity. Finally, how do different SSNs achieve type-specific innervation patterns in response to similar extracellular cues? Neuron type-specific expression of receptors for these cues has been a focus of recent study, but additional mechanisms likely contribute including cell- and context-dependent signal transduction, as well as spatial tuning of receptivity to signals within SSN arbors.

## Author Contributions

CY, EP, JR, and JP wrote this manuscript. All authors contributed to the article and approved the submitted version.

## Conflict of Interest

The authors declare that the research was conducted in the absence of any commercial or financial relationships that could be construed as a potential conflict of interest.
